# Vaginal Pathogenic Bacterial Colonization and Adverse Birth Outcomes among Pregnant Women Living with and without HIV in Tanzania

**DOI:** 10.21203/rs.3.rs-10397690/v1

**Published:** 2026-08-06

**Authors:** Dismas Matovelo, Godfrey W Kajungu, Paul Alikado Sabuni, Michael James, Benson R Kidenya, Moke Magoma, Jeremiah Seni, Jennifer A Downs

**Affiliations:** Catholic University of Health and Allied Sciences; Catholic University of Health and Allied Sciences; Catholic University of Health and Allied Sciences; Catholic University of Health and Allied Sciences; Catholic University of Health and Allied Sciences; Engender Health; Catholic University of Health and Allied Sciences; Weill Cornell Medicine

**Keywords:** Vaginal pathogenic bacteria colonization, HIV, preterm birth, low birthweight, Tanzania

## Abstract

**Background:**

Both preterm birth and low birth weight (LBW) remain major contributors to neonatal morbidity and mortality in sub-Saharan Africa. Vaginal pathogenic bacterial colonization has been implicated in ascending genital tract infections, which may trigger these outcomes. However, there is a paucity of evidence on how vaginal bacterial colonization influences pregnancy outcomes, particularly in the context of maternal HIV infection. This study examined the associations between vaginal colonization by pathogenic bacteria and adverse birth outcomes (preterm birth and LBW) among pregnant women living with HIV and HIV-uninfected pregnant women in Mwanza city, Tanzania.

**Methods:**

A hospital-based prospective analytical cohort study was conducted between August 2023 and February 2025 among pregnant women who delivered at select health facilities in Mwanza city. Eligible women with singleton pregnancies at ≥ 28 weeks gestation were consecutively recruited during the latent phase of labour. Lower vaginal swabs were collected and cultured via standard microbiological methods to identify pathogenic bacteria. Maternal demographic, behavioural, and clinical data were collected through structured questionnaires and clinical assessment tools. The primary outcomes were preterm birth (< 37 weeks) and LBW (< 2500 g). Logistic regression models stratified by HIV status were used to evaluate associations while adjusting for confounders.

**Results:**

A total of 580 pregnant women were enrolled, 70.3% (n = 408) of whom had vaginal pathogenic bacterial colonization. Preterm birth occurred in 14.8% (n = 86) of deliveries, whereas 12.9% (n = 75) had LBW. Infants born to PWLWH had lower mean birth weights than those born to HUPWs did (2.9 kg, 95% CI: [2.8–3.0 kg] vs 3.1 [3.0–3.2] kg; p < 0.001). Overall, vaginal bacterial colonization was not significantly associated with preterm birth or LBW after adjustment for confounding factors among either HUPW (adjusted odds ratio (aOR) 1.13; 95% CI: 0.56–2.27) or PWLWH (aOR 0.76; 95% CI: 0.34–1.72). However, *Escherichia coli* demonstrated nonsignificant trends toward increased odds of LBW in both HUPW (OR = 2.48; 95% CI: 0.81–7.58; p = 0.11) and PWLWH (OR = 2.28; 95% CI: 0.75–6.95; p = 0.15). Vaginal *Staphylococcus aureus* showed a similar trend towards increased odds among PWLWH (OR = 2.76; 95% CI: 0.89–8.59; p = 0.08). Among the 12 (2.1%) ESBL-producing organisms, the odds of preterm delivery among HUPWs were as follows ([Crude odds ratio (COR)]: 11.6, [2.15–62.9; p = 0.004]). No increased odds were observed in PWLWH.

**Conclusion:**

Overall, vaginal colonization was not independently associated with preterm birth or LBW after adjustment for confounders. However, infants of PWLWH had lower mean birth weights, and ESBL-producing bacteria among HUPW may be associated with preterm delivery. ESBL-producing bacteria may be critical and understudied contributors to preterm delivery. Longitudinal studies are needed to inform targeted screening and intervention strategies in pregnancy to address these potentially modifiable factors associated with preterm birth and LBW.

## Introduction

Birth weight is an important indicator of future infant and childhood development(1, 2). Each year, an estimated 15 million babies worldwide are born preterm, and over 20 million are born with low birth weight (LBW), accounting for more than 80% of neonatal deaths(3, 4). Both prematurity and LBW remain major contributors to neonatal morbidity and mortality globally, with the most significant burden occurring in low- and middle-income countries. Sub-Saharan Africa (SSA) bears a disproportionate share of these outcomes due to the high prevalence of maternal infections (HIV, premature rupture of membranes (PROM), limited access to quality and underutilization of antenatal care (ANC), and delays in the diagnosis and treatment of comorbidities such as anaemia, malaria, and hypertension during pregnancy(5, 6). The global average prevalence of preterm birth ranges from 15.3–15.8%, and the prevalence of LBW is estimated at 13.7–16.1%, but the prevalence of LBW in Tanzania is greater. Approximately 16.6% of newborns in Tanzania are born before 37 weeks, and the LBW rate is estimated at 11–18% (7–9).

Vaginal pathogenic bacterial colonization is an essential yet underrecognized contributor to adverse pregnancy outcomes, such as miscarriage, preterm birth, intrauterine growth restriction, stillbirth, and neonatal sepsis(10). The normal vaginal microbiota is typically dominated by *Lactobacillus* species, which inhibit pathogenic organisms by producing lactic acid that lowers the pH and competitively excludes their growth (11, 12). Disruption of this protective microbiota due to behavioral practices or underlying inflammation can lead to overgrowth of potentially pathogenic bacteria (10, 13). Studies have shown that vaginal colonization with organisms such as *Escherichia coli*, *Staphylococcus aureus*, *Group B Streptococcus*, and other gram-negative rods is associated with PROM, urinary tract infections (UTIs), asymptomatic bacteriuria, ascending infection, preterm labour, chorioamnionitis, and early-onset neonatal sepsis(10, 14–17).

Across East Africa, reported vaginal colonization rates range widely from 40% to 75%, depending on population characteristics, laboratory methods, and behavioral practices (15, 18). Certain intravaginal practices, such as douching, have been linked to bacterial imbalance and increased susceptibility to infections, further increasing the risk of PROM and preterm birth(19). With respect to HIV infection, despite the widespread availability of antiretroviral therapy (ART), PWLWH have an elevated risk for preterm birth and LBW, potentially due to persistent immune dysregulation, chronic inflammation, coinfections, and ART-related metabolic effects(1, 20). HIV has also been associated with altered vaginal microbiota composition characterized by reduced *Lactobacillus* dominance and vaginal dysbiosis (12). These changes may amplify susceptibility to ascending infections, increase microbial diversity, and ultimately exacerbate obstetric complications.

Although the burden of infection-related adverse pregnancy outcomes is high, routine ANC in Tanzania rarely includes screening for vaginal pathogenic colonization. Limited laboratory capacity, resource constraints, and inconsistent clinical guidelines contribute to underdiagnosis and missed opportunities for prevention. Evidence on whether and how vaginal colonization interacts with HIV status and behavioral factors to influence adverse outcomes remains scarce, particularly in settings such as Mwanza city and Tanzania, where the HIV prevalence is above the national average and the maternal infection burden is high. Therefore, this study aimed to determine the associations between vaginal pathogenic bacterial colonization, maternal factors, and birth weight and prematurity among a large group of pregnant women living with HIV (PWLWH) and HIV-uninfected pregnant women (HUPW) in Mwanza city, Tanzania.

## Methods

### Study design

This was a hospital-based prospective analytical cohort study conducted from August 2023-February 2025 in selected high-volume labour wards in Mwanza city.

### Study settings

The study was conducted at Bugando Medical Centre (BMC), Sekou Toure Regional Referral Hospital (STRRH), Nyamagana District Hospital (NDH), Buzuruga Health Centre (BHC), and Igoma Health Centre (IHC). Deliveries at the five selected health facilities account for approximately 53% (2,100/4,000) of all institutional deliveries in Mwanza city.

BMC is a teaching and consultant tertiary referral hospital in the lake zone and is located in Mwanza city. It operates as a zonal referral hospital serving a catchment population of 16 million people across eight regions: Kagera, Geita, Mwanza, Shinyanga, Mara, Simiyu, Tabora, and Kigoma. On average, the Labour & Delivery unit performs 450–500 deliveries per month including both low- and high-risk pregnancies. In 2024, approximately 15 pregnant women PWLWH on ARTs and 8–10 newly diagnosed PWLWH received care in the maternity unit. STRRH is a regional hospital in Mwanza city, Nyamagana District. It has 350 beds, including 49 beds in the obstetric ward, handles approximately 400–600 deliveries per month, and serves a catchment population of 2.7 million people. Among all deliveries at the hospital in 2024, approximately 20 were PWLWH. NDH is a district hospital in Nyamagana District, Mwanza city. It serves a catchment population of 1.5 million and performs approximately 450–500 deliveries per month. Among all deliveries in 2024, approximately 10 were PWLWH. The BHC is a health centre in Ilemela district and serves the catchment population of both the Ilemela and Nyamagana districts, with approximately 350–450 monthly deliveries, of whom approximately 15 were PWLWH in 2024. The IHC is a health centre within Nyamagana, serving a catchment population across both the Ilemela and Nyamagana districts, with approximately 250–300 monthly deliveries. Among these, approximately 10 PWLWH were delivered at IHCs in 2024.

### Study population

The study participants were pregnant women who delivered at the labour wards of the five selected health facilities in Mwanza City with a singleton foetus and who had not been referred from another facility. The inclusion criteria were gestational age ≥ 28 weeks in the latent phase of labour (with uterine contractions, cervical effacement, and dilatation of up to 5 cm) and delivery. Pregnant women with multiple pregnancies, vaginal bleeding, cervical cerclage in situ, or referred cases were excluded.

### Sample size and sampling procedure

Sample sizes were calculated via a two-proportional formula (two-proportion z test)(21). n = (Zα/2 + Zβ)^2^ * (p1(1-p1) + p2(1-p2))/(p1-p2), where Zα/2 represents the critical value of the normal distribution at α/2 (for a confidence level of 95%, α = 0.05, and Zα/2 = 1.96), and Zβ represents the critical value of the normal distribution at β (for a power of 80%, β = 0.2, and Zβ = 0.84). P1 was set at 0.1370, and P2 was set at 0.2400(22). The present manuscript reports a secondary analysis examining the association between vaginal pathogenic bacterial colonization and adverse birth outcomes among the recruited cohort on the basis of the parent study, which was powered to compare the prevalence of vaginal pathogenic bacterial colonization between PWLWH and HUPW, with a maximum sample size of 580. In this study, the final sample included sufficient outcome events (86 preterm births and 75 LBW cases) to support the multivariable logistic regression analyses. A consecutive sampling method was employed to recruit eligible patients until the desired sample size was achieved. The sample size for each facility was based on the proportional allocation formula.

### Data collection

Data were collected during the daytime and on weekdays. Upon admission to the maternity ward, ANC cards were reviewed to determine patient eligibility and to confirm gestational age via first-trimester ultrasound, reliable LNMP, or second-trimester ultrasound. Those who were HIV-uninfected during ANC visits were counselled for retesting. A thorough general and obstetrical history and examination were performed on all pregnant women.

Socio-demographic and clinical characteristics, as well as personal behaviours, of study participants were collected using a pre-tested, structured questionnaire developed by the investigators for the study, informed by ANC guidelines and previous studies on vaginal bacterial colonization (Matovelo D et al. Questionnaire. Unpublished; 2023) (Supplementary file 1). To collect information about the presence of possible confounding conditions, participants also underwent testing for malaria via a blood slide, for diabetes mellitus via random blood glucose (women with preexisting diabetes were excluded from GDM testing), for UTIs via urinalysis, and for schistosomiasis via a rapid point-of-care test [POC-CCA3, Mondial Diagnostics, Netherlands]. Haemoglobin levels were also measured to assess anaemia, and hypertensive disorders of pregnancy (HDPs) were diagnosed via blood pressure measurements.

A lower vaginal swab was collected without a speculum before delivery via sterile, prepacked transport medium containing Amies Agar Gel without Charcoal (Copan Diagnostics Inc., Murrieta, CA). The swab was inserted 2–3 cm into the vagina, rotated against the walls for at least 30 seconds, and carefully removed, avoiding contact with the skin and anal area by separating the labia. The vaginal swabs were immediately placed in Amies transport medium in a specimen transport cooler and taken to the CUHAS Multipurpose Laboratory within 2 hours for microbiological analysis. Each sample was cultured via standard methods on blood agar, MacConkey agar (OXOID, UK), and CHROMagarStreB^™^ and incubated at 35°C to 37°C for 18–24 hours.

Bacterial growth in culture media was considered eligible for further biochemical identification tests and AST, taking into consideration that the vagina is a nonsterile site often colonized by microbiota, on the basis of the presence of two criteria: first, the presence of significant growth of at least 10 colony-forming units of one type of bacteria per culture plate; second, the growth of bacterial colonies in at least two quadrants of the culture plate(23). Biochemical identification tests were conducted for gram-positive and gram-negative bacteria via standard methods(24). Antimicrobial susceptibility tests were performed on all identified bacteria via the conventional Kirby–Bauer disk diffusion method on Mueller‒Hinton agar (Oxoid, UK). The respective zones of inhibition or diameters were interpreted as resistant, intermediate, or sensitive using the Clinical Laboratory Standard Institute (CLSI) guidelines for specific management(23).

### Variable Measurement

**Outcome variables**: Preterm birth was defined as delivery before 37 weeks of gestation, and LBW was defined as birth weight < 2,500 grams. **Covariates**: Demographic and clinical characteristics of pregnant women were measured as continuous or categorical variables, depending on their characteristics.

### Data Management and Analysis

The data were entered into Microsoft Excel for cleaning and then imported into Stata 18.0 for analysis. In the descriptive analysis, frequency distributions were used to summarize the demographic characteristics and clinical information of the pregnant women. Continuous variables are reported as the means ± standard deviations, whereas frequencies and proportions were used to summarize categorical variables.

For inferential statistics, logistic regression was used to assess associations between maternal outcomes and vaginal colonization. The study assessed the interaction effects of HIV status, BMI, gestational weight gain, anaemia, and UTIs with each maternal outcome (i.e., premature birth and LBW) via the Mantel–Haenszel method. Confounders were retained in the final model if they altered the estimated association between vaginal pathogenic bacterial colonization and the outcome by at least 10%. Adjusted odds ratios (aORs) and 95% confidence intervals (CIs) are reported. All the statistical analyses were conducted at the 5% significance level.

### Ethical considerations

Ethical clearance was obtained from the joint CUHAS/BMC Research Ethics and Review Committee (CREC/640/2023), National Institute of Medical Research (NIMR) (MR/53/100/731), and Weill Cornell Medicine (26-01029754-02). Permission to conduct this study was sought from the BMC administration, the Mwanza Regional Medical Officer, and the Nyamagana and Ilemela District Councils. This study adhered to the Declaration of Helsinki; all participants aged 18 years and above signed written consent, with a witness present. Parents/guardians signed consent forms for adolescents under 18 years of age, and adolescents signed assent forms. All participants were given an information sheet explaining the purpose and procedures of the study. Illiterate participants provided their thumbprint in the presence of an impartial witness after receiving a thorough explanation of the purpose and procedures orally, with adequate time to ask questions. Participants were assured of the confidentiality of the information collected and that any reports produced would not contain identifying information. Participants were permitted to withdraw from the study, skip responding to questions they deemed uncomfortable or were unwilling to answer, or withdraw consent to participate at any time without consequences.

All women who developed postpartum sepsis were managed in accordance with the hospital sepsis protocol. Women with other pathologies were informed of their results by their treating physicians at the respective health facility and were treated according to standard management guidelines. Any incidental findings were addressed, and participants were assured that these would be interpreted and managed by qualified professionals.

## Results

### Demographic characteristics of the pregnant women

Among the 580 pregnant women (Table 1), the majority were aged 25–35 years (62.2%, n = 361), among whom premature deliveries were (14.4%, n = 52) and LBW infants (13.6%, n = 49). Out-of-pocket healthcare payments were the dominant mode overall (82.9%, n = 481), and this proportion increased further among women with premature (16.2%, n = 78) and LBW (14.3%, n = 69) outcomes. Too little weight gain (< 8 kg) was the most common outcome, affecting 61.9% (n = 359) of women, and was highest among mothers of LBW infants (14.8%; n = 53). Excessive weight gain (> 12 kg) was observed in only 11.4% of the patients (n = 66) (Table 1).

### Clinical characteristics of the pregnant women.

Among pregnant women, the majority had fewer than 8 ANC visits (93.6%, n = 543, overall mean = 4.5 ± 1.7). ANC visits were lower among those who experienced preterm delivery (4.0 ± 1.5, p < 0.001) and LBW (4.2 ± 1.7, p = 0.001). PROM occurred in 8.6% (n = 50) of the patients, with much higher rates among premature (40.0%, n = 20) and LBW deliveries (32.0%, n = 16) (Table 2).

Anaemia affected 35.3% (n = 205) of all women, with a higher prevalence in LBW patients (18.1%, n = 37, p = 0.007). UTIs were present in 21.7% (n = 28, p = 0.013) of premature deliveries and 20.9% (n = 27, p = 0.002) of LBW deliveries. PWLWH tended to have a greater percentage of premature deliveries (18.2%, n = 36, p = 0.102) and significantly more LBW deliveries (23.2%, n = 46, p < 0.001). Colonization remained prevalent (70.3%, n = 408), with similar rates among premature (15.0%, n = 61) and LBW (12.8%, n = 52) deliveries (Table 2).

### Prevalence of LBW and premature birth outcomes among pregnant women

Approximately 12.9% (n = 75, 95% CI: [10.4%–15.9%]) delivered LBW infants. Premature deliveries (< 37 weeks) accounted for 14.8% (n = 86, [12.2%–17.9%]). There was a statistically significant difference in birth weight according to maternal HIV status: infants born to HUPW had a higher mean birth weight (3.1 kg, 95% CI: 3.0–3.2) than those born to PWLWH did (2.9 kg, 95% CI: 2.8–3.0; p < 0.001) (Table 3).

### Assessment of factors associated with vaginal pathogenic bacterial colonization and birth outcomes

A stratified analysis of factors associated with premature delivery revealed minimal confounding (percentage changes below 10%). Interaction assessments indicated no significant effect modification for either preterm or LBW deliveries (p values for homogeneity > 0.05). Antibiotic use was associated with 16.5% lower odds of LBW among HUPW and 0.75% lower odds among PWLWH (Table 4).

### Associations between vaginal colonization and birth outcomes among PWLWH and HUPW.

For preterm delivery, *E. coli* colonization was not significantly associated with increased odds of premature delivery among HUPW (crude odds ratio [COR]: 1.79; [0.79–4.02; p = 0.16]) or PWLWH (COR: 1.36; [0.46–4.03; p = 0.58]). However, ESBL phenotypes were associated with increased odds of premature delivery among HUPW patients ([COR]: 11.6; [2.15–62.9]; p = 0.004). No significant associations between *S. aureus* colonization and premature delivery were detected in HUPW (COR: 0.56; [0.21–1.50; p = 0.25]) or PWLWH (COR: 1.19; [0.38–3.79; p = 0.77]) (Table 5).

Women with *E. coli* colonization showed a nonsignificant trend toward higher odds of having LBW in both HUPW (COR = 2.48; 95% CI: 0.81–7.58; p = 0.11) and PWLWH (COR = 2.28; 95% CI: 0.75–6.95; p = 0.15). Among PWLWH, *S. aureus* was associated with a nonsignificant trend toward increased odds of LBW (COR = 2.76; 95% CI: 0.89–8.59; p = 0.08) (Table 5).

## Discussion

This study contributes to the available evidence on the relationship between vaginal pathogenic bacterial colonization and adverse pregnancy outcomes in a population of women with a high burden of infectious diseases and HIV. In this study, first, vaginal pathogenic bacterial colonization was highly prevalent, affecting more than two-thirds of the participants. Second, overall bacterial colonization was not independently associated with preterm birth or LBW after adjustment for relevant maternal characteristics. Third, species-specific analysis revealed that colonization with *E. coli* and *S. aureus* was associated with increased odds of LBW deliveries, suggesting that specific pathogens, rather than colonization status per se, may lead to infection-related obstetric complications.

The high colonization rates observed in this study are similar to those reported among pregnant women in SSA, which range from 40% to 75%. These high rates likely reflect multiple contextual factors, including hygiene practices, intravaginal cleansing behaviours, sexual practices, antibiotic exposure, and limited access to ANC services, which may influence the vaginal microbiota composition (12, 18). In Tanzania, as in several other LMICs, microbiological screening during ANC is not routinely performed; dysbiosis may remain undetected until complications arise. Despite the high prevalence observed, overall colonization was not independently associated with adverse birth outcomes in this study. This finding suggests that the presence of pathogenic bacteria in the vaginal environment alone may be insufficient to trigger adverse obstetric outcomes. Instead, progression to clinically significant infection may depend on interactions among host, biological, clinical, treatment-related, social, microbial, and obstetric factors, such as immune responses, bacterial virulence, disruption of a protective *Lactobacillus*-dominated microbiota, and ascending infection into the intrauterine environment (10, 25).

The prevalence of preterm birth (14.8%) and LBW (12.9%) observed in this study remains clinically significant and falls within the ranges reported in other tertiary hospitals in Tanzania, Kenya, and Iran (5, 7–9). Women who experienced adverse outcomes had a greater burden of established obstetric and medical risk factors, including anaemia, PROM, hypertensive disorders of pregnancy, current UTIs, HIV infection, and inadequate ANC attendance. These findings are consistent with previous studies demonstrating that maternal comorbidities and health-system factors remain the dominant drivers of adverse pregnancy outcomes, often outweighing isolated microbiological exposures(7, 9).

A key clinical insight from this study is that no effect modification was detected; however, the effect of vaginal pathogenic bacterial colonization on pregnancy outcomes was confounded by antibiotic exposure, particularly among HUPW. Antibiotic exposure likely reflects the presence of other infections or comorbidities, repeated healthcare contact, or microbiome disruption, factors that have been shown to influence pregnancy outcomes(13). In Tanzania, empiric antibiotic use during pregnancy and in the general population is common, brief, and often poorly guided by microbiological evidence, raising concern that such practices may unintentionally increase risk rather than reduce it(26).

Compared with those born to HUPW, infants born to women living with HIV had lower mean birth weights. Importantly, all study participants had been using ART and had preserved CD4 counts. The finding of LBWs aligns with emerging evidence suggesting that HIV exposure in utero may affect fetal growth even in the context of ARTs (1, 20, 27), even though effective ART does reduce several previously observed adverse pregnancy risks associated with HIV, such as mother-to-child transmission, stillbirth, and SGA (28). Persistent immune activation, placental inflammation, and subclinical placental insufficiency due to altered metabolic pathways have been proposed as underlying mechanisms for foetal growth restriction among PWLWH and may not be improved by ART use (20).

In this study, ESBL-producing bacteria among HUPWs were associated with preterm delivery, which is similar to the findings of a previous study conducted in Israel(29). However, nonsignificant trends toward increased odds of LBW with *E. coli* are observed among both HUPW and PWLWH, which is consistent with previous studies suggesting that GNB may be detrimental to pregnancy outcomes. GNB induce endotoxin-mediated inflammatory pathways and possess virulence factors that facilitate adhesion and invasion, contributing to chorioamnionitis, PROM, and intrauterine inflammation, all of which are well-recognized pathways leading to preterm labour and impaired foetal growth (16, 29, 30). In contrast, *S. aureus* was associated with increased odds of LBW in PWLWH. Although *S. aureus* can cause invasive infections, it is a common transient colonizer of mucosal surfaces and has a limited capacity to initiate ascending infection in the absence of additional risk factors (17, 31). These findings imply that pathogen-specific effects and host immune factors need to be evaluated rather than relying on a syndromic approach to assess infection risk during pregnancy. Biologically, ascending infection can trigger intrauterine inflammation via cytokine activation (e.g., IL-6, TNF-α), thereby promoting uterine contractility and fetal growth restriction(30). However, the absence of a significant association in this study may indicate that the detected colonization reflects low-virulence (noninvasive) colonization or is transient, insufficient to elicit a clinically meaningful inflammatory response. Additionally, the PWLWH were immunocompetent, since they were all on ARTs with stable CD counts. Moreover, the lack of association may be due to a lack of microbiome complexity due to reliance on culture.

The findings of this study suggest that routine screening for vaginal colonization alone may have limited predictive value for adverse pregnancy outcomes. Instead, a more targeted approach focusing on pathogen-specific risk assessment may be warranted. In Tanzania and other LMICs, where laboratory resources are limited, these findings support the evaluation of pathogen-specific screening strategies in future interventional studies.

Moreover, the high prevalence of antibiotic exposure among participants underscores the need for improved antimicrobial stewardship during pregnancy. Empirical antibiotic use without microbiological guidance may disrupt the vaginal microbiome and potentially increase susceptibility to pathogenic colonization.

This study has several important strengths. This study included a relatively large sample of pregnant women recruited from multiple high-volume health facilities, enhancing the generalizability of the findings within an urban African context. The use of laboratory-confirmed bacterial cultures allowed objective identification of pathogenic organisms, and stratified analyses by HIV status provided important programmatic insights.

Despite its strengths, this study has several limitations. The study involved single-time sampling, did not evaluate the persistence of colonization and did not determine its acquisition during pregnancy. There was low statistical power in subgroup analysis (ESBL) with respect to organism-specific analysis, with wide confidence intervals indicating imprecision due to the small sample size. Culture-based microbiological methods may not capture the full diversity of the vaginal microbiome, a limitation that could be addressed by molecular sequencing approaches (10). Additionally, factors such as ART adherence, viral load, and other unmeasured behavioural or environmental variables may have influenced the observed associations.

## Conclusion

Overall, vaginal pathogenic bacterial colonization was not independently associated with preterm or LBW deliveries. However, *E. coli* colonization was associated with nonsignificantly increased odds of LBW in both HUPW and PWLWH, whereas *S. aureus* colonization was associated with nonsignificantly increased odds of LBW only among PWLWH, and ESBL-producing bacteria among HUPW were associated with preterm delivery. These findings suggest that pathogen-specific mechanisms may contribute to adverse pregnancy outcomes. These findings support further evaluation of targeted pathogen-specific screening strategies before their routine implementation.

Future studies should employ longitudinal designs to better characterize the dynamics of the vaginal microbiota throughout pregnancy and their relationship with adverse outcomes. Molecular microbiome approaches could provide deeper insights into microbial community structures and host–microbe interactions. Intervention studies evaluating targeted screening and treatment strategies for specific pathogens may also help clarify effective approaches for reducing infection-related pregnancy complications.

## Supplementary Material

This is a list of supplementary files associated with this preprint. Click to download.

SupplementaryFile1Questionnare.pdf

VagColandOutcomesStudydatabaseFINAL.xlsx

## Figures and Tables

**Table 1:** Demographic characteristics of the pregnant women stratified by pregnancy outcomes (N = 580)

Variables		Gestational age at birth			Birthweight		
	Overall n (%)	Term n (%)	Premature n (%)	p value (χ^2^/t test)	Normal n (%)	Low n (%)	p value (χ^2^/t test)
**Age (median, IQR)**	29.5, 25–34	29, 25–34	30, 26–30	0.23	29, 25–34	30, 27–34	0.28
**Age (in years)**				0.68			0.48
≤ 24	123(21.2)	106(86.2)	17(13.8)		111(90.2)	12(9.8)	
25–35	361(62.2)	309(85.6)	52(14.4)		312(86.4)	49(13.6)	
36–44	96(16.6)	79(82.3)	27(17.7)		82(85.4)	14(14.6)	
**Marital status**				0.06			**0.01**
Not married	94(16.2)	74(78.7)	20(21.3)		74(78.7)	20(21.3)	
Married	486(83.8)	420(86.4)	66(13.6)		431(88.7)	55(11.3)	
**Education level**				**0.01**			**0.01**
Primary education	226(39.0)	185(81.7)	41(18.1)		191(84.5)	35(15.5)	
Secondary education	218(37.6)	182(83.5)	36(16.5)		185(84.9)	33(15.1)	
College and above	136(23.4)	127(93.4)	9(6.6)		129(94.9)	7(5.1)	
**Occupation**				0.07			0.24
Not employed	160(27.6)	142(88.8)	18(11.2)		141(88.1)	19(11.9)	
Employed	95(16.40	85(89.5)	10(10.5)		87(91.6)	8(8.4)	
Entrepreneur	325(56.0)	267(82.2)	58(17.8)		277(85.2)	48(14.8)	
**Mode of payment**				**0.04**			**0.03**
Out of pocket	481(82.9)	403(83.8)	78(16.2)		412(85.7)	69(14.3)	
Insurance	99(17.1)	91(91.9)	8(8.1)		93(93.9)	6(6.1)	
**Type of toilet used at home**				0.86			0.27
Western style	523(90.2)	445(85.1)	78(914.9)		458(87.6)	65(12.4)	
Pit latrine	57(9.8)	49(86.0)	8(14.0)		47(82.5)	10(17.5)	
**Wash underpants with disinfectant**				0.47			0.25
No	537(92.6)	459(85.5)	78(14.5)		470(87.5)	67(12.5)	
Yes	43(7.4)	35(81.4)	8(18.6)		35(81.4)	8(18.6)	
**Practice douching**				**0.03**			**0.02**
No	314(54.1)	277(88.2)	37(11.8)		283(90.1)	31(9.9)	
Yes	266(45.9)	217(81.6)	49(18.4)		222(83.5)	44(16.5)	
**Douching frequency/week (n = 266) (Mean, SD)**	13.7, 9.1	13.2, 8.9	15.8, 9.8	0.07	13.1, 9.5	16.6, 9.5	**0.02**
**BMI (mean, SD)**	25.0, 10.3	25.2, 11.0	24.0, 4.7	0.32	25.3, 10.9	23.3, 4.3	0.11
**Weight gain (mean, SD)**	7.2, 4.9	7.2, 4.9	6.8, 4.4	0.58	7.3, 5.0	6.0, 3.6	**0.04**
**Weight gain**				0.56			0.13
Too little (< 8 kg)	359(61.9)	305(85.0)	54(15.0)		306(85.2)	53(14.8)	
Normal (8 kg – 12 kg)	155(26.7)	130(83.9)	25(16.1)		137(88.4)	18(11.6)	
Too much (12 > kg)	66(11.4)	59(89.4)	7(10.6)		62(93.9)	4(6.1)	
**Sex per week (mean, SD)**	2.4, 1.9	2.5, 2.0	2.1, 1.3	0.10	2.5, 1.9	1.9, 1.3	**0.01**
**Area of residence**				**0.03**			0.17
Outside Mwanza city	72(12.4)	55(76.4)	17(23.6)		59(81.9)	13(18.1)	
Within Mwanza city	508(87.6)	439(86.4)	69(13.6)		446(87.8)	62(12.2)	

**Table 2:** Clinical characteristics of the pregnant women stratified by birth outcome (N = 580)

Variables	n (%)	Gestation age at birth			Birthweight		
		Term n (%)	Premature n (%)	p value(χ^2^/t test)	Normal n (%)	Low n (%)	p value(χ^2^/t test)
Gravidity (Mean, SD)	3.2, 1.8	3.1, 1.8	3.5, 1.7	0.04	3.1, 1.8	3.4, 1.8	0.28
Gravidity				**0.03**			0.73
Primigravida	125(21.6)	114(91.2)	11(8.8)		110(88.0)	15(12.0)	
Multigravida	455(78.4)	380(83.5)	75(16.5)		395(86.8)	60(13.2)	
Parity (Mean, SD)	1.8, 1.6	1.8, 1.6	2.1, 1.6	0.067	1.8, 1.6	1.9, 1.6	0.52
Parity				**0.02**			0.30
Nulliparous	156(26.9)	139(89.1)	17(10.9)		137(87.8)	19(12.2)	
Primiparous	111(19.1)	100(90.1)	11(9.9)		101(91.0)	10(9.0)	
Multiparous	313(54.0)	255(81.5)	58(18.5)		267(85.3)	46(14.7)	
ANC visits (Mean, SD)	4.9, 1.7	5.0, 1.7	4, 1.5	**< 0.001**	4.9, 1.7	4.2, 1.7	**0.001**
Below 8 visits	543(93.6)	458(84.4)	85(15.7)	**0.03**	469(86.4)	74(13.6)	**0.05**
8 visits and above	37(6.4)	36(97.3)	1(2.7)		36(97.3)	1(2.7)	
PROM							
No	530(91.4)	464(87.6)	66(12.5)		471(88.9)	59(11.1)	
Yes	50(8.6)	30(60.0)	20(40.0)	**< 0.001**	34(68.0)	16(32.0)	**< 0.001**
Clinical presentations							
Anaemia	205(35.3)	171(83.4)	34(16.7)	0.38	168(82.0)	37(18.1)	**0.007**
History of diabetes mellitus	57(9.8)	48(84.2)	9(15.8)	0.83	48(84.2)	9(15.8)	0.50
Gestational diabetes mellitus (n = 523)^*^	9(1.7)	6(66.7)	3(33.3)	0.11	7(77.8)	2(22.2)	0.38
Malaria	31(5.3)	28(90.3)	3(9.7)	0.41	25(80.7)	6(19.4)	0.27
UTI	129(22.2)	101(78.3)	28(21.7)	**0.01**	102(79.1)	27(20.9)	**0.002**
Preeclampsia	52(9.0)	34(65.4)	18(34.6)	**< 0.001**	33(63.5)	19(36.5)	**< 0.001**
Schistosomiasis	20(3.5)	14(70.0)	6(30.0)	0.05	16(80.0)	4(20.0)	0.33
History of antibiotic use	136(23.5)	105(77.2)	31(22.8)	**0.003**	109(80.2)	27(19.9)	**0.006**
HIV status				0.10			**< 0.001**
Negative	382(65.9)	332(86.9)	50(13.1)		353(92.4)	29(7.6)	
Positive on ARTs Positive not on ARTs	198(34.1) 0	162(81.8) 0	36(18.2) 0		152(76.8) 0	46(23.2) 0	
ART Duration (mean years, SD) (n = 198)	4.5, 4.3	4.1, 4.1	5.9, 4.8	**0.02**	4.2, 4.1	5.4, 4.7	0.09
Colonization status				0.89			0.83
Not colonized	172(29.7)	147(85.5)	25(14.5)		149(86.6)	23(13.4)	
Colonized	408(70.3)	347(85.1)	61(15.0)		356(87.3)	52(12.8)	
Number of pathogens (n = 408)				0.21			0.30
Single	323(79.2)	271(83.9)	52(16.1)		279(86.4)	44(13.6)	
Double	85(20.8)	76(89.4)	9(10.6)		77(90.6)	8(9.4)	
Organism(n = 408)				0.11			0.34
*E. coli*	138(33.8)	110(79.7)	28(20.3)		114(82.6)	24(17.4)	
*S. aureus*	137(33.6)	122(89.1)	15(11.0)		119(86.9)	18(13.1)	
*K. pneumoniae*	32(7.8)	29(90.6)	3(9.4)		31(97.9)	1(3.1)	
*Enterococcus spp*	41(10.1)	35(85.4)	6(14.6)		37(90.2)	4(9.8)	
*P. aeruginosa*	12(2.9)	9(75.0)	3(25.0)		11(91.7)	1(8.3)	
GBS	12(2.9)	11(91.7)	1(8.3)		10(83.3)	2(16.7)	
UGNB	9(2.2)	6(66.7)	3(33.3)		8(88.9)	1(11.1)	
Other GPB	9(2.2)	7(77.8)	2(22.2)		8(88.9)	1(11.1)	
Other GNB	18(4.4)	18(100.0)	0(0.0)		18(100.0)	0(0.0)	
Phenotypes				**0.05**			0.44
None	466(80.3)	399(85.6)	67(14.4)		407(87.3)	59(12.7)	
MRSA	68(11.7)	60(88.2)	8(11.8)		60(88.2)	8(11.8)	
MRSA+iMLSB	32(5.5)	27(84.4)	5(15.6)		26(81.3)	6(18.8)	
ESBL	12(2.1)	7(58.3)	5(41.7)		11(91.7)	1(8.3)	
iMLSB	2(0.3)	1(50.0)	1(50.0)		1(50.0)	1(50.0)	

* Only women without known diabetes underwent testing

*Hints*: GBS: *Group B Streptococcus*, GPB: gram-positive bacteria, UGNB: Unidentified gram-negative bacteria, Other GPB: *Streptococcus* spp., Other GNB: (*K. oxytoca, Acinetobacter* spp., *Aeromonas hydrophilia, Citrobacter* spp., *K. aerogenes, Proteus* spp., *Shigella* spp., *and Enterobacter* spp.).

**Table 3:** Prevalence of LBW and premature birth among pregnant women (N = 580)

Variable	n (%)	95%CI	HIV Status			Colonization Status		
			Uninfected (N = 382) n (%)	Infected (N = 198) n (%)	p value (χ^2^/t test)	Not colonized (N = 172) n (%)	Colonized (N = 408) n (%)	p value(χ^2^/t test)
Birth-weight (Mean, SD)	3.0	2.9–3.1	3.1, 0.5	2.9, 0.6	**< 0.001**	3.0, 0.5	3.0, 0.5	0.99
Normal birthweight (> = 2.5 kg)	505(87.1)	84.15–89.6%	353(69.9)	152(30.1)	**< 0.001**	149(29.5)	356(70.5)	0.83
Low birthweight (< 2.5 kg)	75(12.9)	10.4%-15.9%	29(38.7)	46(61.3)		23(30.7)	52(69.3)	
Category low birthweight (n = 75)								
Low (1.5 kg-2.4 kg)	67(89.4)	79.9%-94.6%	25(37.3)	42(62.7)	0.43	22(32.8)	45(67.2)	0.47
Very low (1 kg-1.4 kg)	7(9.3)	4.4%-18.5%	3(42.6)	4(57.1)		1(14.3)	6(85.7)	
Extremely low (< 1 kg)	1(1.3)	0.0%-9.1%	1(100)	0(0.0)		0(0.0)	1(100.0)	
Gestation age at birth (Mean, SD)	38.4, 2.3	38.2–38.5	38.4, 2.3	38.3, 2.4	0.93	38.4, 2.4	38.4, 2.3	0.87
Post term (Above 42 weeks)	19(3.3)	2.1%-5.1%	9(47.4)	10(52.6)	**0.046**	6(31.6)	13(68.4)	0.97
Term (37–42 weeks)	475(81.9)	78.5%-84.8%	323(68.0)	152(32.0)		141(29.7)	334(70.3)	
Premature (< 37 weeks)	86(14.8)	12.2%-17.9%	50(58.1)	36(41.9)		25(29.1)	61(70.9)	
Category of premature birth (n = 86)								
Premature later term (34–36 weeks)	64(74.4)	64.0%-82.6%	36(56.3)	28(43.2)	0.26	18(28.1)	46(71.9)	0.19
Premature moderate term (32–33 weeks)	9(10.5)	5.5%-19.1%	4(44.4)	5(55.6)		1(11.1)	8(88.9)	
Premature very preterm (28–32 weeks)	13(15.1)	8.9%-24.5%	10(79.9)	3(23.1)		6(46.2)	7(523.8)	

**Table 4:** Stratified analysis for assessment of confounders in the association between vaginal pathogenic bacteria colonization and birth outcomes

Premature delivery									
Variables	Overall population		HUPW		PWLWH	
	Interaction assessment	Confounder assessment		Interaction assessment	Confounder assessment		Interaction assessment	Confounder assessment	
	p-value for homogeneity	AOR_1_	% change	p-value for homogeneity	AOR_2_	% change	p-value for homogeneity	AOR_3_	% change
HIV status	0.56								
BMI	0.62			0.59			0.28		
Weight gain	0.75			0.91			0.56		
Anaemia	0.41			0.35			0.60		
UTI	0.19			0.14			0.86		
Education level		1.01	2.34		1.11	4.35		0.86	0.90
History of antibiotic use		0.95	8.23		1.05	9.07		0.80	5.41
Previous history of genital infection		1.05	1.60		2.56	1.19		0.83	1.68
LBW									
Variables	Overall population		HUPW		PWLWH	
	Interaction assessment	Confounder assessment		Interaction assessment	Confounder assessment		Interaction assessment	Confounder assessment	
	p-value for homogeneity	AOR_1_	% change	p-value for homogeneity	AOR_2_	% change	p-value for homogeneity	AOR_3_	% change
HIV status	0.78								
BMI	0.46			0.61			0.67		
Weight gain	0.31			0.26			0.81		
Anaemia	0.99			0.93			0.82		
UTI	0.53			0.37			0.55		
Education level		0.93	2.20		0.94	4.08		0.85	0.96
History of antibiotic use	0.87	8.21			0.82	**16.45**			0.84	0.75
Previous history of genital infection	0.95	0.50			1.01	2.25			0.84	0.71

**Note**: *Respectively crude association COR_1_=1.03, COR_2_=1.16, COR_3_=0.85*

**Note**: *Respectively crude association COR_1_=0.95, COR_1_=0.97, COR_2_=0.84*

**Table 5:** Association between vaginal pathogenic bacterial colonization and birth outcomes with HIV status.

Premature delivery						
Variables	HUPW (Model 1)			PWLWH (Model 2)		
	aOR*_1_*	95% CI	p value	aOR*_2_*	95% CI	p value
**Colonization status**						
Not colonized	1			1		
Colonized	1.13	0.56–2.27	0.73	0.76	0.34–1.72	0.51
Variables^*^	HUPW (Model 1)			PWLWH (Model 2)		
	COR*_1_*	95% CI	p value	COR*_2_*	95% CI	p value
**Organisms**						
Others	1			1		
*E. coli*	1.79	0.79–4.02	0.16	1.36	0.46–4.03	0.58
*S. aureus*	0.56	0.21–1.50	0.25	1.19	0.38–3.79	0.77
**Phenotype (MRSA)**						
No	1			1		
Yes	0.44	0.12–1.59	0.209	0.43	0.09–2.14	0.305
**Phenotype (ESBL)**						
No	1			1		
Yes	11.62	2.15–62.90	**0.004**	0.96	0.09–10.05	0.976
Low birthweight						
Variables	HUPW (Model 1)			PWLWH (Model 2)		
	aOR*_1_*	95% CI	p value	aOR*_2_*	95% CI	p value
**Colonization status**						
Not colonized	1			1		
Colonized	0.84	0.35–1.97	0.68	0.82	0.39–1.72	0.61
Variables^*^	HUPW (Model 1)			PWLWH (Model 2)		
	COR*_1_*	95% CI	p value	COR*_2_*	95% CI	p value
**Organisms**						
Others	1			1		
*E. coli*	2.48	0.81–7.58	0.11	2.28	0.75–6.95	0.15
*S. aureus*	1.02	0.29–3.66	0.97	2.76	0.89–8.59	0.08
**Phenotype (MRSA)**						
No	1			1		
Yes	0.57	0.11–3.03	0.508	0.81	0.19–3.43	0.777
**Phenotype (ESBL)**						
No	1			1		
Yes	2.1	0.21–21.04	0.528	1		

* Species-specific analyses were exploratory and unadjusted because of sparse data.

**Note:** *Crude association prior to adjustment: COR_1_=1.16, COR_2_=0.85; All models were adjusted by education, history of antibiotic use, and history of genital infection*

**Note:** *Crude associations: COR_1_=0.97, COR_2_=0.84; All models were adjusted by education, history of antibiotic use, andhistory of genital infection*

## Data Availability

The dataset supporting the conclusions of this article is included as an additional file.

## Abbreviations

ANC: Antenatal Care
AMR: Antenatal Resistance
ARTs: Antiretroviral therapies
BMC: Bugando Medical Centre
BHC: Buzuruga Health Centre
CLSI: Clinical Laboratory Standard Institute
CTC: Care & Treatment Centre
CUHAS: Catholic University of Health & Allied Sciences
GNB: Gram Negative Bacteria
HDP: Hypertensive Disorders of Pregnancy
HICs: High-income Countries
HUPW: HIV-uninfected Pregnant Women
HIV: Human Immunodeficiency Syndrome
IHC: Igoma Health Centre
IRR: Incidence Rate Ratio
IUGR: Intrauterine Growth Restriction
LBW: Low birthweight
L&D: Labour & Delivery
NDH: Nyamagana District Hospital
LMICs: Low-Middle Income Countries
PWLWH: Pregnant Women Living With HIV
SGA: Small for Gestation Age
SSA: Sub-Saharan Africa
STRRH: Sekou Toure Regional Reflection Hospital
UTIs: Urinary Tract Infections
